# The Association of Fatty Liver and Diabetes Risk

**DOI:** 10.2188/jea.13.15

**Published:** 2007-11-30

**Authors:** Masako Okamoto, Yasuhisa Takeda, Yoshioki Yoda, Kazuhisa Kobayashi, Masayuki A. Fujino, Zentaro Yamagata

**Affiliations:** 1 Department of Health Sciences, Yamanashi Medical University.; 2 Yamanashi Kouseiren Medical Center.; 3 First Department of Medicine, Yamanashi Medical University.

**Keywords:** fatty liver, diabetes, hyperglycemia

## Abstract

We examined whether fatty liver, as diagnosed with abdominal ultrasonography, is an independent risk factor for diabetes mellitus during 10 years of follow-up. A total of 840 subjects (467 men and 373 women) were followed for the entire 10 years. The criteria for being non-diabetic were having no history of diabetes, having a fasting plasma glucose level of less than 110 mg/dl and a serum hemoglobin A_1c_ level of 6.4% or less. We indicated that every examine received all examinations after 12 hours of fasting. Well-trained technicians performed abdominal ultrasonography. Although univariate analysis revealed that the presence of fatty liver was related to hyperglycemia 10 years later, multiple logistic regression analysis did not support this finding. In the multiple logistic regression analysis fasting plasma glucose levels at the baseline and age were significantly related to hyperglycemia (odds ratio [OR] = 1.16, 95% confidence interval [CI]: 1.11-1.21, OR = 1.07, 95% CI: 1.01-1.14, respectively). Fatty liver was not an independent risk factor for hyperglycemia in our follow-up study 10 years after the first diagnosis. The high fasting plasma glucose levels were a risk factor for diabetes, even in the normal range.

Recently, the prevalence of diabetes mellitus and related deaths have increased in Japan. The 1997 Ministry of Health and Welfare investigation estimated that there were about 13,700,000 patients with diabetes in Japan, of which only about 6,900,000 had been diagnosed.^1^ Many of the patients were type 2 diabetes mellitus, and the importance of preventive care has been emphasized. Type 2 diabetes mellitus is mainly associated with obesity; however, it has also been shown to be associated with a fatty liver.

A fatty liver or steatohepatitis is the most common liver disease^2^ and its incidence has increased recently in Japan,^3^ especially in those between the ages of 40 and 50 years.^4^ Although the etiology of fatty liver and nonalcoholic steatohepatitis (NASH) are not completely known, studies indicate that steatohepatitis and fatty liver are related to obesity,^3^^,^^5^^-^^19^ dyslipidemia,^5^^,^^8^^,^^9^^,^^12^^-^^14^^,^^16^^-^^18^^,^^20^ type 2 diabetes mellitus,^8^^-^^11^^,^^13^^,^^17^^,^^20^^,^^21^ leptin,^5^^,^^15^ and insulin resistance.^16^^-^^19^^,^^20^^-^^24^ Most of these studies were case-control and cross-sectional studies. A cohort study suggested that serum *γ*-glutamyltransferase (*γ*-GTP) level was an independent risk factor for non-insulin dependent diabetes mellitus (NIDDM).^25^ To our knowledge, however, no cohort studies have examined the association between a fatty liver, as diagnosed on ultrasonography, and diabetes risk. Therefore, we examined whether the diagnosis of fatty liver, based on ultrasonographic results, is a risk factor for diabetes over a 10-year follow-up period.

## METHODS

A total of 2653 subjects (1491 men and 1162 women) voluntarily visited Yamanashi Kouseiren Medical Center for general check-ups from April 1, 1991 through March 31, 1992.

All the subjects lived in Yamanashi Prefecture, a rural area located about 100 km west of Tokyo. The subjects included agricultural workers, white-collar workers, and business people. All the study participants filled in a self-administered questionnaire about their smoking habits (cigarettes per day, the number of years of smoking), alcohol consumption (kinds, quantity, the number of years of alcohol intake), and present and past medical histories and family medical history. The questionnaires were checked by 2 public health nurses in the center.

Five well-trained technicians performed abdominal ultrasonographic examinations. These technicians had no information about the present illness, or laboratory test results of the patients. A 3.5-MHz mechanical sector-type probe (Aloka 650, 680, 2000, Tokyo, Japan) was used. All ultrasonographic images were recorded on videotape, and images of any abnormal abdominal findings were also recorded on instant film for later reviews by technicians and physicians. After the examination was complete, both the technician who conducted the examination and another technician reexamined the videotape to check for abnormal findings. Furthermore, a physician checked any abnormal findings and conducted follow-up examinations.

The ultrasonographic diagnostic criteria for fatty liver were made according to the modified criteria of Kurtz et al.^26^ These criteria include the presence of diffusely increased parenchymal echogenicity (bright liver), which is associated with an unusually fine liver texture, the increased attenuation of the ultrasound beam, and the decreased visualization of hepatic and portal veins. Subjects without any of these findings were classified as not having fatty liver.

Case records of all examinees were entered into a computer database (TOSHIBA EQUIUM, Tokyo, Japan). All the records were available and complete.

Anthropometric data (body weight and height) were measured simultaneously (Tanita, Tokyo, Japan). Body mass index (BMI) (kg/m^2^) was calculated and served as an index for overall obesity. Serum levels of total cholesterol (TC), triglycerides (TG), high-density lipoprotein cholesterol (HDL-C), aspartate aminotransferase (AST), alanine aminotransferase (ALT), *γ*-GTP, fasting plasma glucose (FPG), and hemoglobin A_1c_ (HbA_1c_) were examined using an auto-analyzer. We indicated that every examine receiving all examinations was after 12 hours of fasting.

Subjects were divided into 2 groups according to followed criteria: the normal was defined as FPG level <110 mg/dl and HbA_1c_ of 6.4% or less. If the FPG was equal or >110 mg/dl or the HbA_1c_ was >6.4%, it was considered as hyperglycemia. The normal range of HbA_1c_ in our institution is 6.4% or less. The normal range of FPG was according to World Health Organization (WHO) criteria.^27^ The 75-g oral glucose tolerance test was not included in our routine health examination.

**Figure 1.**
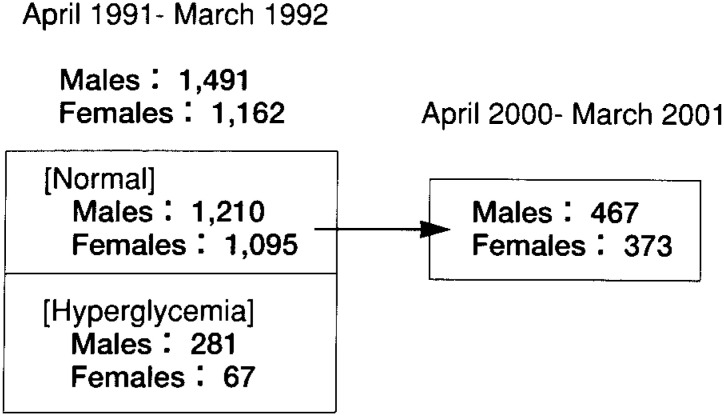
The number of subjects with normal blood glucose levels and hyperglycemia who were examined from April 1, 1991 through March 31, 1992, and the number of those who underwent a follow-up examination between April 1, 2000 and March 31, 2001.

Among the 2653 subjects examined between April 1, 1991 and March 31, 1992, 2305 (1210 men and 1095 women) had normal FPG and HbA_1c_ levels and no history of diabetes. Among these subjects, 840 (467 men, 373 women) underwent a follow-up examination between April 1, 2000, and March 31, 2001 (Figure 1). Many of these subjects received regular check-ups every 1 or 2 years. The frequency of check-ups during the 10 years of follow up was also examined.

Data are expressed as mean ± standard deviation (SD). The following statistical tests were applied as appropriate: Mantel-Haenszel estimates of odds ratios (OR) with 95% confidence intervals (CI); unpaired Student’s t test; chi-squared test with Fisher’s exact test; and multiple logistic regression analysis. A 2-tailed *P* < 0.05 significance level was selected for all analyses. All statistical analyses were performed using the Statistical Analysis System version 6.12 (SAS Institute Inc., Cary, North Carolina, USA).

## RESULTS

A total of 2305 of 2653 subjects had normal FPG and HbA_1c_ levels at baseline (from April 1, 1991 through March 31, 1992). Among the subjects with normal values, we compared body constitution and laboratory results of the 840 subjects who underwent follow up examinations 10 years later (from April 1, 2000 through March 31, 2001) (group A) with the 1465 subjects who did not undergo follow-up examinations 10 years later (group B). The prevalence of fatty liver was not significantly different between these 2 groups (men: group A, 22.8%, group B, 25.1%; women: group A, 5.1%, group B 7.9%). Among women, values for FPG (90.2 ± 9.5 vs 92.3 ± 15.7; *P* = 0.047), TC (177.1 ± 31.4 vs 181.0 ± 43.6; *P* = 0.046), and TG levels (74.7 ± 40.0 vs 82.1 ± 43.6; *P* = 0.049) were significantly lower in group A than in group B, respectively (Table 1).

**Table 1.** Comparison of baseline data without hyperglycemia who did (group A) and did not (group B) undergo follow-up examinations 10 years later.

sex	males		p value	female		p value
	Group A	Group B		Group A	Group B	
	(n=467)	(n=743)		(n=373)	(n=722)	
fatty liver (%)	22.8%	25.1%	0.374^a^	5.1%	7.9%	0.095^a^
Age (yr)	42.3 ± 4.7	42.2 ± 4.5	0.553	43.0 ± 4.1	43.1 ± 3.9	0.550
FPG (mg/dl)	97.4 ± 13.6	98.5 ± 18.2	0.189	90.2 ± 9.5	92.3 ± 15.7	0.047
HbA1c (%)	6.0 ± 0.6	6.0 ± 0.6	0.727	5.7 ± 0.4	5.7 ± 0.6	0.124
BMI (kg/m^2^)	22.9 ± 2.7	23.1 ± 2.8	0.310	22.1 ± 2.7	22.2 ± 2.8	0.819
AST (IU/L)	21.7 ± 11.9	22.8 ± 11.3	0.060	16.4 ± 5.0	16.4 ± 5.6	0.788
ALT (IU/L)	28.1 ± 27.0	29.2 ± 20.5	0.388	14.8 ± 8.0	15.1 ± 10.1	0.645
*γ*-GTP (IU/L)	43.1 ± 45.2	47.4 ± 51.3	0.092	12.2 ± 8.4	13.9 ± 11.8	0.095
TC (mg/dl)	184.4 ± 32.0	186.3 ± 32.8	0.275	177.1 ± 31.4	181.0 ± 43.6	0.046
TG (mg/dl)	134.3 ± 93.5	139.2 ± 93.5	0.328	74.7 ± 40.0	82.1 ± 43.6	0.049
HDL-C (mg/dl)	50.9 ± 13.7	50.7 ± 14.0	0.805	60.0 ± 13.1	59.3 ± 12.9	0.405

A group: the examiners without hyperglycemia who received April 1, 1991through March 31, 1992 and April 1, 2000 through March 31, 2001.

B group: the examiners without hyperglycemia who received April 1, 1991through March 31, 1992 and not received April 1, 2000 through March 31, 2001.

FPG, fasting plasma glucose; HbA_1c_, hemoglobin A_1c_;

BMI, body mass index; AST, aspartate aminotransferase; ALT, alanine aminotrasferase;

*γ*-GTP, *γ*-glutamyltransferase; TC, serum total cholesterol; TG, triglycerides;

HDL-C, high density lipoprotein cholesterol

^a^ *χ*^2^ test

Table 2 shows the number of men and women with and without fatty liver at baseline and hyperglycemia at the end of follow-up. After 10 years of follow-up, 66 of 467 men (14.1%) and 16 of 373 women (4.3%) had hyperglycemia. The prevalence of hyperglycemia at the end of follow-up increased 2.62 fold (95 % CI: 1.58-4.34) over baseline as shown by univariate analysis. The prevalence of fatty liver increased 10.66 fold (95 % CI: 7.02-16.21) at the end of follow-up compared with baseline.

**Table 2.** Association of ultrasonographic findings at baseline and hyperglycemia at the end of follow-up.

	ultrasonographic findings	normal	hyperglycemia	total	OR (95% CI)
Men	Non-fatty liver	325	42 (4)	367	1
	Fatty liver	76	24 (5)	100	2.44(1.41-4.23)

Women	Non-fatty liver	340	13 (0)	353	1
	Fatty liver	17	3 (0)	20	4.62(1.34-15.87)

Men and Women	Non-fatty liver	665	55 (4)	720	1
	Fatty liver	93	27 (5)	120	2.62(1.58-4.34)

OR: odds ratio, CI: confidence interval

The number of diabetic pattern at the end of follow-up in parentheses.

The body constitution and serum analysis at baseline, frequency of medical check-ups, alcohol drinking habits, and a family history of diabetes in subjects with fatty liver and non-fatty liver are shown in Table 3. At baseline, there were no significant differences in age, the frequency of medical check-ups, or the ratio of BMI changes between the 2 groups. For both men and women, respectively, signficantly higher values were seen in the fatty liver group compared with the non-fatty liver group in terms of BMI (*P* = 0.001 and *P* < 0.001), AST (*P* = 0.002 and *P* = 0.019), ALT (*P* = 0.001 and *P* < 0.001), TG (*P* = 0.001 and *P* = 0.037), and FPG (*P* = 0.005 and *P* = 0.009). Significantly lower levels of HDL-C were seen in the non-fatty liver group than the fatty liver group for both men (*P* = 0.001) and women (*P* < 0.001). Among men, *γ*-GTP (*P* = 0.016), TC (*P* < 0.001), and HbA_1c_ (*P* = 0.005) levels were significantly higher in the fatty liver group than in the non-fatty liver group. There were no significant differences for men or women in the alcohol drinking habits and the family history of diabetes between the 2 groups. Similarly, there were no significant differences in the amount of alcohol consumption for drinkers in both groups (data not shown). There were no significant differences in the frequency of the medical check-ups, or the ratio of BMI changes during the ten years between the 2 groups.

**Table 3.** Comparison between fatty liver group and non-fatty liver group.

characteristic	Men		p value	Women		p value
	Fatty liver	Non-fatty liver		Fatty liver	Non-fatty liver	
	(n=100)	(n=367)		(n=20)	(n=353)	
Age(yrs)^#^	41.9 ± 4.9	42.1 ± 4.7	0.767	43.9 ± 4.0	42.8 ± 4.1	0.270
BMI (kg/m^2^)^#^	24.9 ± 2.2	22.2 ± 2.3	0.001	26.1 ± 3.1	21.9 ± 2.6	<0.001
Δ BMI^†^	1.3 ± 5.3	2.3 ± 5.7	0.109	0.66 ± 5.4	1.83 ± 6.8	0.460
AST (IU/L) ^#^	24.5 ± 9.8	20.1 ± 10.5	0.002	21.0 ± 7.8	16.2 ± 4.8	0.019
ALT (IU/L) ^#^	36.4 ± 20.6	23.9± 24.8	0.001	26.5 ± 14.3	14.2 ± 7.0	<0.001
*γ*-GTP (IU/L) ^#^	53.3 ± 52.6	39.4 ± 43.4	0.016	15.3 ± 7.5	11.9 ± 8.3	0.073
TC(mg/dl) ^#^	193.9 ± 36.0	179.9 ± 30.3	<0.001	184.4 ± 33.4	175.6 ± 30.8	0.228
TG (mg/dl) ^#^	182.4 ± 105.9	116.9 ± 77.0	0.001	122.0 ± 72.0	72.0 ± 34.2	0.037
HDL-C (mg/dl) ^#^	44.4 ± 11.2	53.1 ± 13.9	0.001	49.3 ± 12.6	60.4 ± 12.9	<0.001
FPG (mg/dl) ^#^	96.3 ± 6.9	94.0 ± 7.2	0.005	93.4 ± 9.3	88.8 ± 7.4	0.009
HbA1c (%) ^#^	5.9 ± 0.3	5.8 ± 0.4	0.005	5.8 ± 0.5	5.6 ± 0.4	0.099
frequency ^§^	8.3 ± 2.4	8.5 ± 2.2	0.121	8.4 ± 2.1	8.2 ± 2.2	0.68
drinking habits ^‡^^#^	72.0%	79.4%	0.131^a^	30.0%	24.5%	0.611^a^
family history ^¶^	11.7%	6.9%	0.133^a^	9.4%	5.2%	0.706^b^

^#^ baseline data

BMI, body mass index at baseline, AST, aspartate aminotransferase; ALT, alanine aminotrasferase; *γ*-GTP, *γ*-glutamyltransferase; TC, serum total cholesterol; TG, triglycerides; HDL-C, high density lipoprotein cholesterol; FPG, fasting plasma glucose; HbA_1c_, hemoglobin A_1c_

† Δ BMI= (BMI at the end of follow-up-BMI at the baseline)/BMI at the baseline × 100

§ frequency: frequency of medical check-ups during this ten years

‡ drinking habits: ratio of persons who have drinking habits

¶ family history: the presence of diabetes of their parents

^a^ *χ*^2^ test, ^b^ Fisher’s exact test

Fatty liver was not significantly associated with hyperglycemia (OR = 1.83, 95% CI: 0.95-3.51) after adjusting for sex, age, BMI, FPG, and HbA_1c_ at baseline, BMI changes, frequency of examinations, alcohol drinking habits, and family history of diabetes (Table 4). Similar results were seen on multiple regression analysis by sex (males: OR = 1.67, 95% CI: 0.81-3.43, females: OR = 3.83, 95% CI: 0.75-19.47). FPG levels at baseline and age were significantly related to hyperglycemia 10 years later (OR = 1.16, 95% CI: 1.11-1.21, OR = 1.07, 95% CI: 1.01-1.14, respectively) in multiple logistic regression analysis adjusted for fatty liver and the other factors listed above.

**Table 4.** Odds ratios (OR) and 95 % confidence intervals (CI) for hyperglycemia ( IFG or DM) according to other factors.

	adjusted OR (95 % CI)
Fatty liver	1.83 (0.95-3.51)
Sex (female/male)	0.63 (0.30-1.29)
Age	1.07 (1.01-1.14)
BMI	1.08 (0.97-1.20)
Δ BMI^†^	1.03 (0.98-1.09)
FPG	1.16 (1.11-1.21)
HbA_1c_	1.42 (0.64-3.14)
frequency ^§^	1.13 (0.98-1.30)
alcohol drinking habits ^‡^	1.11 (0.59-2.10)
family history ^¶^	1.47 (0.62-3.36)

BMI, body mass index;

FPG, fasting plasma glucose; HbA_1c_, hemoglobin A_1c_;

† Δ BMI= (BMI at end of follow-up-BMI at baseline) /BMI at baseline × 100

§ frequency: number of medical check-ups during this 10-year follow-up

‡ alcohol drinking habits:yes/no

¶ family history: presence of diabetes in parents

Men who did not drink alcohol or who drank alcohol less than 40 g/week were looked at to examine the relationship between non-alcoholic fatty liver and diabetes. There are 20 cases of hyperglycemia in the group of 136 non-diabetic men. There was a significant association between the fatty liver group and hyperglycemia (OR = 4.45, 95% CI: 1.64-12.1). However, no significant association was seen in multiple logistic regression analysis (OR = 1.85, 95% CI: 0.40-8.51).

Among non-obese males (BMI of 24.2 or less at both baseline and the end of follow-up), FPG levels at baseline were related to hyperglycemia 10 years later (OR = 1.22, 95% CI: 1.15-1.30) as shown by multiple logistic regression analysis.

## DISCUSSION

Our study shows that fatty liver is associated with hyperglycemia among apparently healthy people followed over a 10-year period (using univariate analysis). These results are in agreement with previous studies,^8^^-^^11^^,^^13^^,^^17^^,^^20^^,^^21^ although these studies were case-control or cross sectional studies. However, using multiple logistic regression analysis adjusting for age, BMI, FPG levels, and HbA_1c_ levels at baseline, BMI changes, drinking habits, parental diabetes, and frequency of check-ups, no association was observed. Thus it does not appear that the presence of fatty liver is an independent risk factor for hyperglycemia.

Obesity, aging, lack of exercise, genetic factors, and stress are known as risk factors of type 2 diabetes mellitus.^28^ In terms of obesity and fatty liver, previous studies have shown that fatty liver occurred more frequently in obese subjects than in normal-weight subjects.^3^^,^^5^^-^^19^ The BMI levels were significantly higher in the fatty liver group than in the non-fatty liver group in our study. Although there was not difference in age between fatty liver group and non-fatty liver group, Nomura^4^ showed that the prevalence of fatty liver was higher in 40s than 30s of age. We think that these factors lead to the diminution of the association of fatty liver and hyperglycemia over time.

Our study should be interpreted carefully, however, because of the limitations in the study design. First, our population sample consisted of people who voluntarily decided to have the examinations. They may be more concerned with their health than people who have not received physical check-ups. We do not know how many people have diabetes or fatty liver in our area because no investigation of these people was performed. From this reason, we guessed that the prevalence of hyperglycemia in our study was less than that of the general population in our prefecture. Second, the number of subjects in group A represented about one third of subjects. The members of this group may be particularly concerned with their health or have other health problems. There were no significant differences between groups A and B in terms of the prevalence of fatty liver. However, among women, FPG, TC, and TG levels were significantly lower in the group A than in group B. It is thought that some of the women in group B in this study were diagnosed with diabetes and went to other clinics, so they did not receive a follow-up examination 10 years later. This may be one of the reasons that the prevalence of fatty liver among women in this study was low. The results of multiple regression analysis showed the same results among men and women. Finally, the men and women who were diagnosed with fatty liver had consultations with doctors and nurses. Therefore, there is a high possibility that they changed their eating and exercise habits, which could have influenced the prevalence of diabetes. Thus, the relation between fatty liver and diabetes in our study may be underestimated.

Previous studies^16^^-^^19^^,^^22^^-^^24^ reported that there were associated with insulin and hepatic insulin resistance even in the absence of diabetes in subjects with steatohepatitis, fatty liver, and NASH. Perry et al^25^ performed a cohort study and reported that raised *γ*-GTP levels are an independent risk factor for NIDDM. Given the results of previous studies, we suspected that fatty liver leads to future diabetes. Our results showed that fatty liver is associated with diabetes, but is not an independent risk factor for diabetes.

In terms of the diagnosis of fatty liver, a liver biopsy is necessary to precisely diagnose as fatty liver and determine the types of fatty liver diseases. Invasive procedures were not allowed in the present study. Abdominal ultrasonograms are noninvasive and an easy method for examining liver conditions and other organs. A previous study indicated that ultrasonographic detection and quantitative hepatic fat accumulation are similar with computed tomography and liver biopsy.^29^ A previous study in which ultrasonographic findings were compared with histologic results also indicated that the overall sensitivity and specificity of ultrasonographic examinations for the diagnosis of fatty liver are approximately 80% to 95% and 90% to 95%, respectively.^30^ Many names have been applied to the same condition. Ludwig et al^11^ shows the terminology for steatohepatitis and a classification of NASH.

The results of this study show that FPG levels at baseline were related to hyperglycemia after 10 years, even when FPG levels were in the normal range. FPG levels were also associated with diabetes among men with normal weight and no diabetes. Men who had high FPG levels, even with normal weight, should be careful to maintain a lifestyle to prevent diabetes. We cannot explain about women in detail because the number of women in our study who suffered from diabetes was small.

It is not clear what kinds of people with fatty liver and steatohepatitis are at high risk for diabetes in the future. Further investigations including genetic factors are required.
